# Generation of a human iPSC-derived cardiomyocyte/fibroblast engineered heart tissue model

**DOI:** 10.12688/f1000research.139482.1

**Published:** 2023-09-27

**Authors:** Max J Cumberland, Jonas Euchner, Amar J Azad, Nguyen T N Vo, Paulus Kirchhof, Andrew P Holmes, Chris Denning, Katja Gehmlich

**Affiliations:** 1 Institute of Cardiovascular Sciences, University of Birmingham, Birmingham, England, B15 2TT, UK; 2 Centre of Membrane Proteins and Receptors, University of Birmingham, Birmingham, England, B15 2TT, UK; 3 Biodiscovery Institute, University of Nottingham, Nottingham, England, NG7 2RD, UK; 4 Department of Cardiology, University Heart and Vascular Center Hamburg, Universitat Hamburg, Hamburg, Hamburg, 20251, Germany; 5 Institute of Clinical Sciences, University of Birmingham, Birmingham, England, B15 2TT, UK; 6 Division of Cardiovascular Medicine, Radcliffe Department of Medicine, University of Oxford, Oxford, England, OX3 9DU, UK

**Keywords:** Engineered Heart Tissue (EHT), 3D cardiac model, induced pluripotent stem cell derived cardiomyocytes (iPSC-CM), induced pluripotent stem cell derived cardiac fibroblasts (iPSC-CF), cardiac co-culture, cardiac fibrosis

## Abstract

Animal models have proven integral to broadening our understanding of complex cardiac diseases but have been hampered by significant species-dependent differences in cellular physiology. Human-induced pluripotent stem cell-derived cardiomyocytes (hiPSC-CMs) have shown great promise in the modelling of cardiac diseases despite limitations in functional and structural maturity. 3D stem cell-derived cardiac models represent a step towards mimicking the intricate microenvironment present in the heart as an *in vitro* model. Incorporation of non-myocyte cell types, such as cardiac fibroblasts, into engineered heart tissue models (EHTs) can help better recapitulate the cell-to-cell and cell-to-matrix interactions present in the human myocardium. Integration of human-induced pluripotent stem cell-derived cardiac fibroblasts (hiPSC-CFs) and hiPSC-CM into EHT models enables the generation of a genetically homogeneous modelling system capable of exploring the abstruse structural and electrophysiological interplay present in cardiac pathophysiology. Furthermore, the construction of more physiologically relevant 3D cardiac models offers great potential in the replacement of animals in heart disease research. Here we describe efficient and reproducible protocols for the differentiation of hiPSC-CMs and hiPSC-CFs and their subsequent assimilation into EHTs. The resultant EHT consists of longitudinally arranged iPSC-CMs, incorporated alongside hiPSC-CFs. EHTs with both hiPSC-CMs and hiPSC-CFs exhibit slower beating frequencies and enhanced contractile force compared to those composed of hiPSC-CMs alone. The modified protocol may help better characterise the interplay between different cell types in the myocardium and their contribution to structural remodelling and cardiac fibrosis.

Research highlights **Scientific benefits**

• Generation of a more physiologically relevant human cardiac model
• Utility as a genetically homogenous system for disease modelling of cardiac arrhythmia, heart disease and cardiomyopathy
• Capacity in exploring cellular dynamics integral to cardiac fibrosis

**3Rs benefits**

• Engineered heart tissue models can replace the use of small animal models of cardiac arrhythmia, cardiomyopathy and heart failure
• Acquiring data from the small animal models used in cardiovascular research often requires the animal to undergo multiple moderate severity procedures which require repeated anaesthesia, such as electrocardiograms and mini pump/telemeter implantation
• As mouse physiology is vastly different to human hearts, insights from mouse models cannot always be extrapolated to human hearts. In contrast, engineered heart tissue models use human cells with human physiology
• Many of the pathogenic variants modelled in mouse lines cause chronic and severe illness

**Practical benefits**

• The relatively low cost in comparison to mouse models
• Induced pluripotent stem cells are relatively easier to genetically manipulate than *in vivo* models
• Ability to generate healthy human quiescent cardiac fibroblasts
• Reproducible methods for the derivation of cardiomyocytes and cardiac fibroblasts from hiPSCs
• Accessibility of cardiac cells with a consistent genome

**Current applications**

• Disease modelling of cardiac arrhythmia, cardiomyopathy and heart failure
• Exploring physiological interplay between cardiac fibroblasts and cardiomyocytes

**Potential applications**

• Low throughput drug screening platform for cardiotoxicity
• Use as a human model to investigate the reversibility of cardiac fibrosis

## Introduction

Limited availability of healthy human heart tissue combined with its inherent nature as a non-proliferative tissue type has meant that heart disease researchers have historically been reliant on *in vivo* models. Large animal models used in heart disease research, for example dogs, pigs, and sheep, are broadly analogous to humans in cardiac physiology and anatomy but are heavily restricted by cost, throughput, and the sentiment of the general public ( Camacho *et al*., 2016). Small animal models commonly used in cardiovascular research, such as the mouse and zebrafish, offer a cheaper but drastically less physiological and anatomical alternative. Zebrafish offer distinct advantages including optical transparency and rapid development but are limited in their capacity as a cardiac model by their two-chambered heart and temperature-dependent action potential ( Vornanen and Hassinen, 2016). Significant differences in the intracellular electrophysiology of mouse cardiomyocytes, caused by contrasting expression and activation of the delayed rectifier and transient outward K ^+^ currents as well as the voltage-gated sodium and calcium channels, are broadly illustrated in their comparatively rapid heart rate of between 500-700 bpm ( Xu *et al*., 1999; Niwa and Nerbonne, 2010; Blechschmidt *et al*., 2008). Consequently, the modelling of cardiac diseases such as arrhythmia, cardiomyopathy and heart failure, which often manifest through concomitant electrical and structural remodelling, is often hampered in mice due to distinct species-dependent differences.

The co-development of human-induced pluripotent stem cell (hiPSC) technology and CRISPR-Cas 9 genome editing provides a new type of genetically modified disease model based on human cells ( Ma *et al*., 2018; Mosqueira *et al*., 2018). The utility of induced pluripotent stem cell-derived cardiomyocytes (hiPSC-CM) has caused a shift in the approach many cardiovascular researchers take to cardiac disease modelling, with many groups opting to replace or supplement the use of small animal models with comparatively cheaper stem cell-based models ( Cumberland *et al*., 2022). Consequently, hiPSC-CM have been used to successfully model pathogenic variants that predispose individuals to cardiac diseases, including arrhythmia and cardiomyopathy ( Lin *et al*., 2015; Goktas Sahoglu *et al*., 2022; Jung *et al*., 2022; Reyat *et al*., 2020; Schulz *et al*., 2023).

The incorporation of hiPSC-CM into cardiovascular disease modelling has however not come without difficulty. Early attempts at differentiating cardiomyocytes produced variable and electrophysiologically immature cells with depolarized diastolic membrane potentials, slow action potential upstroke velocities and large pacemaker currents ( Knollmann, 2013). Optimisation and development of differentiation protocols enabled the derivation of chamber-specific cardiomyocytes to explore diverse disease aetiologies but offered marginal advancements in variability and maturity ( Jeziorowska *et al*., 2017; Devalla *et al*., 2015). Accordingly, innovative maturation strategies were developed to increase the maturity of the cells produced. Current methods include: micropatterning, metabolic maturation, electrical stimulation, soft-substrate culture, co-culture, and engineered 3D models ( Jimenez-Vazquez *et al*., 2022; Feyen *et al*., 2020; Yoshida *et al*., 2018; Tzatzalos *et al*., 2016; Machiraju and Greenway, 2019).

With greater electrophysiological, metabolic, structural, and functional maturity, 3D cardiac models offer greater potential in understanding the nuances of complex cardiac diseases such as heart failure and cardiomyopathy ( Correia *et al*., 2018; Shadrin *et al*., 2017; Ulmer *et al*., 2018; Vučković *et al*., 2022). Furthermore, the concurrent ease in which contractile force and transmembrane potential can be monitored in 3D cardiac models presents great potential for their future incorporation into *in vitro* cardiotoxicity studies ( Gintant *et al*., 2019). A number of 3D cardiac models have been generated to examine cardiac physiology in disease including, but not limited to: two-post engineered heart tissues (EHTs), ring-shaped engineered heart muscle (EHM), cardiac patches, cardiac micro-, and biowires, and Novoheart ( Tiburcy *et al*., 2017; Ronaldson-Bouchard *et al*., 2018; Zhang *et al*., 2013; Thavandiran *et al*., 2013; Zhao *et al*., 2019). Although differing substantially in size and shape, all the 3D cardiac models listed above attempt to better recapitulate the cell-to-cell and cell-to-matrix interactions present in the myocardium and encourage the longitudinal alignment of cardiomyocytes upon a scaffold ( Smith *et al*., 2017).

Despite occupying the majority of the volume of the mammalian heart, cardiac myocytes are estimated to account for less than 50 % of the total cell number ( Zhou and Pu, 2016). Major non-myocyte cell types in the heart include cardiac fibroblasts and endothelial cells as well as ancillary populations of immune cells and autonomic neurones. Therefore, the incorporation of non-myocyte cell populations into cardiac modelling systems should facilitate the structural, metabolic, and electrophysiological maturity of hiPSC-CM and aid in generating a more physiologically relevant microenvironment ( Yoshida *et al*., 2018; Kim *et al*., 2010). Up to now, cell types commonly incorporated into 3D cardiac models include endothelial cells, mesenchymal stem cells, and cardiac fibroblasts ( Tadano *et al*., 2021; Tulloch *et al*., 2011; Zhang *et al*., 2017).

Cardiac fibroblasts are a common cell type in human ventricles (circa 20 % by number, [ Pinto *et al*., 2016]) and are integral to the architecture, alignment, and electromechanical properties of the myocardium in health and disease. Quiescent or inactive induced pluripotent stem cell-derived cardiac fibroblasts (hiPSC-CF) can be derived from hiPSCs and used effectively as a stand-alone model of cardiac fibrosis ( Zhang *et al*., 2019). The trans-differentiation of the quiescent cardiac fibroblast to myofibroblast occurs prior to and during the development of cardiac fibrosis and is often difficult to prevent *in vitro* as the cells cultured on plastic dishes are subject to a Young’s modulus up to a million times stiffer than the native myocardium ( Landry *et al*., 2019; Acevedo-Acevedo and Crone, 2015). This pathological transition is substantially stimulated by the profibrotic signalling cytokine TGF-β1. Suppression of TGF-β1 can be achieved *in vitro* using small molecule inhibitors such as SB 431542 and can be used on *in vitro* models to control the activation status of quiescent cardiac fibroblasts ( Law and Carver, 2013).

An amalgamated 3D cardiac model consisting of hiPSC-CF and hiPSC-CM presents the opportunity for the generation of genetically homogenous (isogenic) systems capable of investigating the pathophysiological interplay present in cardiac diseases such as cardiomyopathy and arrhythmia. Here we describe a modified protocol for the generation and incorporation of quiescent hiPSC-CF into an engineered heart tissue model.

In this study we describe efficient protocols for the differentiation of hiPSC-CM and quiescent cardiac fibroblasts. Methods are outlined detailing the incorporation of these cells into EHTs with improved contractile function and tissue compaction and potential use exploring the pathophysiological interplay between hiPSC-CM and hiPSC-CF in cardiac fibrosis. In the UK, 51,427 procedures were carried out on mice in 2021 for research on the cardiovascular, blood and lymphatic system (Home Office Report on Annual Statistics of Scientific Procedures on Living Animals Great Britain 2021). Assuming that 5 % of these mice were used to study cardiac arrhythmia, heart failure or cardiomyopathy, use of the model proposed could lead to the direct replacement of 2500 mice per year in the UK alone.

## Methods

### Reagents for hiPSC culture and differentiation

### hiPSC culture

Induced pluripotent stem cells Kolfc2 (WTSIi018-B) were maintained on six-well plates coated with Geltrex (1:100; for details of reagents and suppliers see Table 1) according to the manufacturer’s protocol. Cells were cultured in mTeSR Plus media at 37 °C and 5 % CO _2_ and passaged at 70-80 % confluency. Cells were passaged by removing the media in the well, washing once with PBS and adding TrypLE Express Enzyme (1X), phenol red for 3 minutes at 37°C. The TrypLE Express was removed and the cells were gently washed off the surface of the well with 1 mL of warm mTeSR Plus medium containing 10 μM Rock Inhibitor (Y-27632). Cells were passaged according to a seeding density of 20,000 cells per cm ^2^ and cultured in 10 μM Rock Inhibitor (Y-27632) mTeSR Plus media for the first 24 hours before switching to mTeSR Plus. The Rho-kinase inhibitor (Rock Inhibitor), Y-27632 was added to prevent dissociation-induced apoptosis. It should be removed 24 hours after the passaging of the cells to maintain iPSC pluripotency.

**Table 1.** Reagents for hiPSC culture and differentiation.

Reagent and preparation	Company and catalogue number
Kolfc2 (WTSIi018-B)	https://ebisc.org/WTSIi018-A
mTeSR Plus	StemCell Technologies, 100-0276
DPBS, no calcium, no magnesium	ThermoFisher Scientific, 14190144
Corning Costar 6-well Clear TC-treated	Fisher Scientific, 10578911
Corning 25 cm ^2^ cell culture flask TC-treated rectangular canted neck	Sigma-Aldrich, CLS430639-200EA
Geltrex LDEV-Free Reduced Growth Factor Basement Membrane Matrix	ThermoFisher Scientific, A1413201
TrypLE Express Enzyme (1X), phenol red	ThermoFisher Scientific, 12605010
TrypLE Select Enzyme (1X), no phenol red	ThermoFisher Scientific, 12563011
Rock Inhibitor Y-27632 (Dihydrochloride) ( *Reconstituted in PBS to 10 mM and stored at -20 °C in 50 μL aliquots*)	StemCell Technologies, 72304
StemPro-34 SFM (1X)	ThermoFisher Scientific, 10639011
L-Glutamine (200 mM)	ThermoFisher Scientific, 25030081
Recombinant Human BMP-4 Protein ( *Reconstituted in 4 mM HCl (0.1 % BSA) to 50 μg/mL and stored at -80 °C in 5 μL aliquots*)	R&D Systems, 314-BP-010/CF
Human Activin A Recombinant Protein ( *Reconstituted in PBS (0.1 % BSA) to 10 μg/mL and stored at -20 °C in 20 μl aliquots*)	ThermoFisher Scientific, PHC9564
RPMI 1640 Medium	ThermoFisher Scientific, 11875093
B-27 Supplement, minus insulin	ThermoFisher Scientific, A1895601
XAV 939 ( *Reconstituted in DMSO to 10 mM and stored at -20 °C in 25 μL aliquots*)	TOCRIS, 3748
KY 02111 ( *Reconstituted in DMSO to 10 mM and stored at -20 °C in 25 μL aliquots*)	TOCRIS, 4731
B-27 Supplement (50X), serum free	ThermoFisher Scientific, 17504044
Retinoic Acid ( *Reconstituted in DMSO to 50 mM and stored at -80 °C in 5 μL aliquots*)	Sigma-Aldrich, R2625-50MG
RPMI 1640 Medium, no glucose	ThermoFisher Scientific, 11879020
CHIR 99021 ( *Reconstituted in DMSO to 10 mM and stored at -20 °C in 10 μl aliquots)*	TOCRIS, 4423
IWR-1 ( *Reconstituted in DMSO to 10 mM and stored at -20 °C in 10 μL aliquots)*	Sigma-Aldrich, I0161
Accutase solution	Sigma-Aldrich, A6964
Advanced DMEM/F-12	ThermoFisher Scientific, 12634010
Fibroblast Growth Medium 3	(PromoCell, C-23130)
Recombinant Human FGF basic/FGF2/bFGF (146 aa) Protein ( *Reconstituted in PBS (0.1 % BSA) to 10 μg/mL and stored at -80 °C in 50 μL aliquots)*	bio-techne, 233-FB-010
SB 431542 ( *Reconstituted in DMSO to 10 mM and stored at -80 °C in 50 μL aliquots)*	TOCRIS, 1614

### hiPSC-CM differentiation

**Figure 1.**
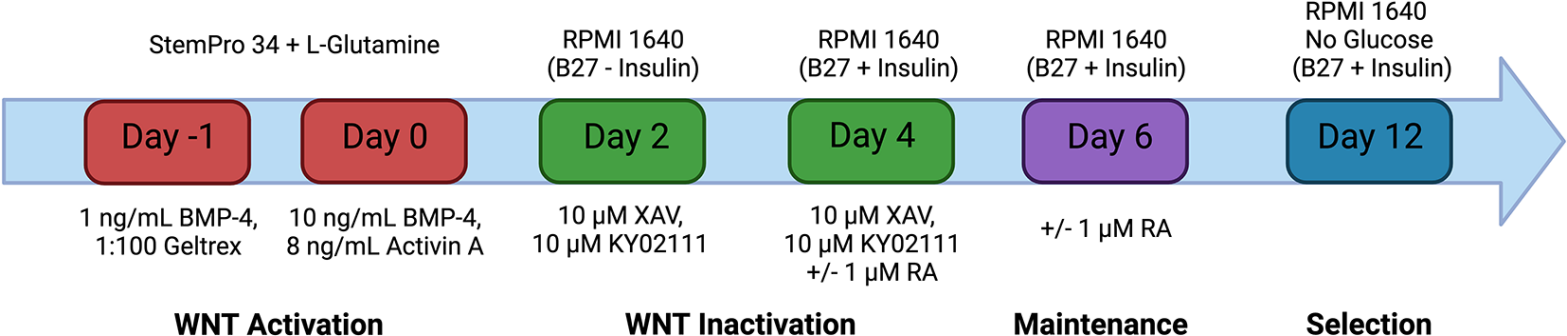
Differentiation of hiPSC-CM. Schematic representation of the hiPSC-CM differentiation protocol. Mesodermal specification is achieved through the activation of the Wnt signalling pathway using the recombinant proteins: Bone morphogenic protein- 4 (BMP-4) and Activin A. Subsequent inactivation of the Wnt signalling pathway by small molecules XAV 939 and KY02111 enables the differentiation of cardiac progenitor cells into induced pluripotent stem cell derived cardiomyocytes. Differentiation can be driven towards atrial cardiomyocyte specification through the addition of retinoic acid on days 4 and 6 only. The medium on the cells was changed every 48 hours after day 6 with RPMI 1640 with B27 + Insulin. Selection is achieved from days 12-14 by culturing the cells in RPMI 1640 No Glucose with B27 + insulin for 48 hours. Created with Biorender.com.

The method for differentiation of hiPSC-CM was broadly adapted from the protocol outlined in Smith *et al*. (2018) ( Figure 1). hiPSCs were seeded onto six-well plates coated with Geltrex and cultured in 2 mL per well of mTeSR Plus. The medium was changed on the cells every 48 hours until the cells reached 60 % confluency. The medium was then changed for 24 hours with StemPro-34 SFM (1X) supplemented with 2 mM L-Glutamine, 1 ng/mL Recombinant Human BMP-4 Protein and 1:100 Geltrex. The cells were subsequently changed with StemPro medium supplemented with 10 ng/mL BMP-4, 8 ng/mL Activin A and 2 mM L-Glutamine and incubated for 48 hours (day 0). The medium was changed with RPMI 1640 Medium with B-27 Supplement, minus insulin, 10 μM XAV 939 and 10 μM KY 02111 for 48 hours (day 2). The cells were subsequently changed with RPMI 1640 Medium with B-27 Supplement (50X), 10 μM XAV 939 and 10 μM KY02111 for 48 hours (day 4). The medium on the cells was changed with RPMI 1640 Medium with B-27 Supplement (50X) every other day. Atrial differentiation was achieved through the addition of 1 μM retinoic acid to the media on days 4 and 6. At 12 days after the initiation of differentiation, glucose starvation was performed to purify the population of cells for cardiomyocytes. This was achieved by changing the medium of the cells with RPMI 1640 Medium supplemented 1:50 with no glucose with B-27 Supplement (50X) for 48 hours. Cells were dissociated at day 15 for incorporation into EHT models. This media was removed from the well(s), the well was washed once with 1 mL of PBS and the cells were incubated in 2 mL of TrypLE Select 10× for 30 minutes at 37 °C. The cells were then washed from the surface of the plate with pre-warmed DMEM-F12.

### hiPSC-CF differentiation

**Figure 2.**
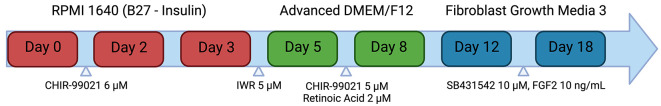
Differentiation of hiPSC-CF. Schematic representation of the hiPSC-CF differentiation protocol. Mesodermal specification is achieved through the activation of the Wnt signalling pathway using CHIR-99021. Subsequent inactivation of the Wnt signalling pathway by the small molecule IWR1 allows the generation of cardiac progenitor cells. Culture in CHIR-99021 and retinoic acid drives the cells towards the derivation of cardiac fibroblasts. Cardiac fibroblasts are maintained in medium supplemented with FGF2 and the TGF-β1 inhibitor SB 431542 to prevent activation into myofibroblasts. Created with Biorender.com.

The method for the differentiation of quiescent induced pluripotent stem cell-derived cardiac fibroblasts was broadly adapted from the protocol outlined in Zhang *et al*. (2019) ( Figure 2). Induced pluripotent stem cells (Kolfc2/WTSIi018-B) were seeded at 30,000 cells/cm ^2^ in a T25 flask pre-coated with 1:100 Geltrex for 2 hours at 37 °C, in 10 μM Rock Inhibitor (Y-27632) mTeSR Plus medium. The cells were cultured for 48 hours prior to the medium being changed with RPMI 1640 supplemented with B27 minus insulin and 6 μM CHIR-99021 for 48 hours. The medium was then changed with RPMI 1640 supplemented with B27 minus insulin for 24 hours. The cells were subsequently treated with a RPMI 1640 with B27 minus insulin and 5 μM IWR1 for 48 hours. The cells were dissociated by washing with 3 mL PBS and then treating with 5 mL accutase enzyme for 3 minutes at 37 °C. The cells were removed from the flask, diluted with 5 mL pre-warmed RPMI 1640 and spun down at 200 g for 3 minutes. The cells were resuspended in Advanced DMEM/F-12 supplemented with 5 μM CHIR-99021 and 2 μM Retinoic Acid and seeded at 20,000 cells/cm ^2^ in a Geltrex coated T25 flask. The cells were incubated in this medium for 3 days and then subsequently changed to Advanced DMEM/F-12 for a further 4 days. The cells were passaged and plated into a Geltrex coated T25 flask at a density of 20,000 cells/cm ^2^ in Fibroblast Growth Medium 3 supplemented with 10 ng/mL FGF2 and 10 μM of the TGF-β1 inhibitor SB431542. The cells were cultured in this media for 6 days, with media changes taking place every 48 hours. Following the 6 days, the cells can be assessed for purity using immunocytochemistry. It is recommended that the cells are expanded, passaged at 70-80 % confluency for 2 passages and cryopreserved for use in future experiments in serum free freezing medium. Cells were passaged by washing once with PBS, and then incubating in TrypLE™ Express for 3 minutes at 37 °C. Cells can be dislodged from the surface of the flask by gently tapping the sides of the container. Following the detachment of the cells, a 2 × volume of DMEM/F12 was added to dilute the TrypLE. The TrypLE/cell mix was centrifuged at 300 g for 3 minutes. The cells were resuspended in Fibroblast Growth Medium 3 supplemented with 10 ng/mL FGF2 and 10 μM SB431542 and seeded at a density of 10,000 cells/cm ^2^.

### hiPSC-derived EHTs

**Protocol for EHT construction**

Here we describe a step-by-step protocol for the construction of four EHTs (one strip) containing iPSC-CF. The reagents required are listed in Table 2.

**Table 2.** List of reagents and suppliers, preparation and storage.

Reagent/Media	Preparation and storage
Silicon Rack (DiNAQOR, C0001)	Autoclave and keep sterile.
Teflon Spacer (DiNAQOR, C0002)	Autoclave and keep sterile.
UltraPure Agarose (ThermoFisher Scientific, 16500100)	Dissolve agarose in sterile PBS to obtain a 2 % agarose solution. Autoclave and store at 4 °C.
10x DMEM, powder, high glucose (ThermoFisher Scientific, 12100061)	Dissolve 1.34 g in 10 mL of sterile Tissue-Culture H _2_O and sterilise by passing through a 0.22 μm syringe filter (Merck Millex™-GP Sterile Syringe Filter, 10268401). Store at 4 °C for no longer than 1 week.
33 mg/mL Aprotinin (Sigma-Aldrich, A1153-100MG)	Dissolve 100 mg in 3.03 mL of sterile H _2_O. Filter sterilise, divide into 50 μL aliquots and store at -20 °C.
200 mg/mL Fib-Ap/Fibrinogen-Aprotinin Mix (Sigma-Aldrich, F8630-1G)	Gradually add 1 g of fibrinogen to 5 mL pre-warmed 0.9 % NaCl H _2_O. *NOTE: Fibrinogen is difficult to dissolve in H _2_O. Add small quantities of fibrinogen to the 0.9 % NaCl H _2_O, warm in the incubator and place on the roller to ensure complete dissolution.* Add 14.42 μl of aprotinin (33 mg/mL). Divide into 50 μL aliquots and store at -80 °C.
100 U/mL Thrombin (Sigma-Aldrich, T4648-1KU)	Dissolve thrombin (1000 U) in 6 mL PBS and 4 mL H _2_O. Divide into 50 μl aliquots and store at -80 °C.
5 mL of 2X DMEM (Make up fresh each time)	• 1 mL of 10x DMEM • 1 mL Gibco Horse Serum, heat inactivated, New Zealand origin (ThermoFisher Scientific, 11540636) • 100 μL Penicillin-Streptomycin (ThermoFisher Scientific, 11548876) • 2.9 mL sterile Tissue-Culture H _2_O Sterile filter. Do not store.
Rock Inhibitor Y-27632 (Dihydrochloride) (Stem cell Technologies, 72304)	• Reconstitute in PBS to 10 mM, divide into 50 μL aliquots and store at -20 °C
NKM Medium	• 100 mL DMEM, high glucose (ThermoFisher Scientific, 11965084) • 10 mL FBS (Sigma-Aldrich, F9665-50ML) • 1 mL Penicillin-Streptomycin (ThermoFisher Scientific, 11548876) • 1 mL L-Glutamine (ThermoFisher Scientific, 25030081) Store at 4 °C for no longer than 1 week.
EHT Medium	• 45 mL DMEM, high glucose (ThermoFisher Scientific, 11965084) • 500 μL Penicillin-Streptomycin (ThermoFisher Scientific, 11548876 • 50 μL Insulin (10 mg/mL) • (Merck Millipore, 91077C-250MG) • 50 μL Aprotinin (33 mg/mL) • (Sigma-Aldrich, A1153-100MG) Filter sterilise and store at 4°C *Add 5 mL of horse serum (ThermoFisher Scientific, 11540636) to 45 mL of above medium prior to use. The medium can be supplemented with 10 μM of the TGF-β1 inhibitor SB 431542 (TOCRIS, 1614) to prevent cardiac fibroblast activation.*
Nunc Cell-Culture Treated 24 well plate (ThermoFisher Scientific, 142475)	• Other brands of 24 well plate have differing dimensions and will reduce success rate of EHT formation
TrypLE Select Enzyme (10X) (ThermoFisher Scientific, A1217701)	• Single cell dissociation is required for the generation of a homogenous EHT
0.5 ml TubeOne Microcentrifuge Tubes, Natural (non-sterile) (StarLabs, S1605-0000)	• Autoclave sterilised and pre-chilled.

### Preparation of reagents for EHT construction

1. Ensure there is premade sterile 2 % agarose in the fridge
2. Prepare and filter sterilise 2X DMEM mix, and leave on ice
3. Ensure there is premade NKM medium and chill on ice
4. Ensure there is premade EHT medium
5. Prewarm 40 mL of sterile DMEM-F12 in the water bath

### Dissociation of hiPSC-CM

1. Remove media and wash 1-2 confluent wells of a six-well plate containing iPSC-CM with room temperature PBS once
  NOTE: 1 × confluent well of hiPSC-CM in a 6 well plate should yield approximately 4 × 10 ^6^ cells
2. Add 2 mL of TrypLE Select Enzyme (10X) per well of a 6 well plate and incubate for 30 minutes at 37 °C
3. Transfer the detached cells to a 50 mL centrifuge tube and wash off any attached cells with 1 mL per well of pre-warmed DMEM-F12
4. Add DMEM-F12 to the cell mix to give a total volume of 10 mL
  NOTE: Centrifugation of iPSC-CM takes place later, when both cell types have been combined
5. Perform a cell count

### Dissociation of hiPSC-CF (performed during Step 2 of hiPSC-CM dissociation)

1. Remove the media and wash a T25 flask (80 % confluency) containing iPSC-CF with PBS
  NOTE: A T25 flask at 80 % confluency should yield approximately 600,000 iPSC-CF
2. Add 3 mL TrypLE Express and incubate for 3 minutes at 37 °C
3. Gently tap the flask on all sides to dissociate cells from the surface of the flask
4. Transfer the 3 mL of TrypLE/cell mix to a 50 mL centrifuge tube
5. Rinse the flask with 7 mL of pre-warmed DMEM-F12 before combining it with the TrypLE containing cells
6. Perform a cell count

### Combining cells and centrifugation

1. Combine required volumes of hiPSC-CM (4 × 10 ^6^ cells) and hiPSC-CF (6 × 10 ^5^ cells) cell mixes in a centrifuge tube and make up to 30 mL with pre-warmed DMEM-F12
2. Spin down the tube containing both iPSC-CM and iPSC-CF at 100 g for 10 minutes
3. Place thrombin, Fib-Ap, Rock Inhibitor and Geltrex on ice
4. Following centrifugation, gently place the cells on ice
  NOTE: Be careful not to disturb the pellet

### Making agarose moulds (performed during 10-minute centrifugation)

1. Microwave 2 % agarose until completely molten
2. Pipette 1.6 mL of molten agarose per well in a column of a Nunc 24 well plate
3. Insert a Teflon spacer into a column of the Nunc 24 well plate containing molten agarose to create agarose moulds for a silicon rack of four EHTs
  NOTE: Wells of a 24 well plate can differ in dimensions. The spacers and silicon racks are optimised for use in Nunc Cell-Culture Treated 24 well plates
4. Remove the Teflon spacers from the now solidified agarose moulds after 15 minutes
  NOTE: Agarose moulds can deteriorate and crack over time. Cast EHTs in the agarose moulds 10 minutes after removal of the Teflon spacers
5. Place the silicon racks into the moulds, ensuring central alignment
6. NOTE: Misaligned silicon racks increase the risk of snagging during removal from the moulds

### Preparing the EHT mix (See Table 3)

**Table 3.** EHT Mix composition.

Component	1X	4.5X (Mastermix for 4 EHTs)
**1: NKM Medium**	81.9 μl	368.55 μl
**2: 200 mg/mL Fib-Ap Mix**	2.5 μl	11.25 μl
**3: 2X DMEM** (made up fresh)	5.5 μl	24.75 μl
**4: Rock inhibitor (10 mM)**	0.1 μl	0.45 μl
**5: Geltrex**	10 μl	45 μl

1. Aliquot 3 μL of the thrombin into 4 × 0.5 mL microcentrifuge tubes and chill on ice (4 tubes for 4 EHTs)
  NOTE: Briefly spin down the tubes containing the thrombin
2. Add 368.55 μL of NKM medium to a prechilled 1.5 mL microcentrifuge tube
3. Prewarm the Fib-Ap mix in your hands (to enable accurate pipetting) and then add 11.25 μL to the NKM medium
  NOTE: Complete dissolution of the Fib-Ap mix is paramount to successful generation of EHTs.
4. Add 24.75 μL of 2X DMEM
5. Add 0.45 μL of Rock Inhibitor
6. Add 45 μL of geltrex
7. Resuspend the cell pellet containing both hiPSC-CM and hiPSC-CF in the EHT mix
  NOTE: Everything should be kept on ice at this stage.

### Casting EHTs

1. Add 100 μL of the EHT cell mix to a 3 μL aliquot of thrombin and then quickly pipette the mix in between the two silicon posts resting inside the agarose mould
  NOTE: Minimise risk of air bubble formation by only pipetting down to the first stop of the pipette
2. Repeat step 1 for each EHT
  NOTE: Be careful not to knock or disturb the 24 well plate holding the solidifying EHTs
3. Gently triturate the EHT cell mix intermittently (every 4 EHTs) to ensure homogeneity
4. Gently place the lid on the 24 well plate and place in a 37 °C incubator for 2 hours

### Removal and culture of EHTs

1. Warm NKM medium in a 37 °C water bath
2. Add 1.5 mL of EHT medium to each well of a column of wells in a Nunc 24 well plate and place in the 37 °C incubator
3. After 2 hours, gently pipette 350 μL of prewarmed NKM medium on top of the agarose mould in each well
  NOTE: Pipetting on top of the agarose mould prevents disruption to the EHT
4. Place in the incubator for a further 10 minutes
5. Move the plate from side to side to encourage dislodging of the EHTs
6. Carefully but firmly remove the EHT strips from the agarose moulds and place into the wells containing the prewarmed EHT medium
  NOTE: Removal of the strips should be performed in a single action as prolonged and gentle removal of the EHTs from the agarose moulds can increase the risk of snagging and malformation
8. Maintain the EHTs by transferring the silicon racks into wells containing fresh EHT medium every 48 hours.

### RT-qPCR

hiPSC-CF were cultured in T25 flasks for 72 hours in Fibroblast Growth Media 3 supplemented with 10 ng/mL FGF2 + 10 μM SB 431542 or 10 ng/mL FGF2 + 10 ng/mL TGF-β1 (R&D Systems, 240-B-002/CF). RNA extraction was performed using the Direct-zol RNA Miniprep Kit (Zymo Research, R2050) according to the manufacturer’s protocol. cDNA was generated using the High-Capacity cDNA Reverse Transcription Kit (ThermoFisher Scientific, 4368814) according to the manufacturer’s protocol. RT-qPCR was performed using TaqMan Fast Advanced Master Mix (ThermoFisher Scientific, 4444557) following the manufacturer’s protocol and on a QuantStudio 5 Real-Time PCR System, 384-well (ThermoFisher Scientific, A28140). RT-qPCR was performed using TaqMan Gene Expression Assay (FAM) (ThermoFisher Scientific, 4331182) for *ACTA2* (Hs00426835_g1) and *COL1A1* (Hs00164004_m1). All reactions were multiplexed and normalised to the housekeeping gene *GAPDH* using Human *GAPD* ( *GAPDH*) Endogenous Control (VIC/MGB probe, primer limited) (ThermoFisher Scientific, 4326317E). 10 ng of cDNA were used per reaction. Transcript levels were measured from three samples of iPSC-CF, with each sample being obtained from cells at different passages. Cycle threshold values were obtained from three technical replicates of each sample. Relative gene expression was calculated using the comparative cycle threshold method ( Schmittgen and Livak, 2008).

### Immunofluorescence

*Induced pluripotent stem cell derived cardiac fibroblasts*

hiPSC-CF were split into wells of a Geltrex-coated 24 well plate (10,000/well) and cultured for 72 hours in Fibroblast Growth Media 3 supplemented with 10 ng/mL FGF2 + 10 μM SB 431542 or 10 ng/mL FGF2 + 10 ng/mL TGF-β1. Cells were fixed for 14 minutes at room temperature using 4 % paraformaldehyde Solution (ThermoFisher Scientific, J19943.K2). Fixed cells were blocked for 1 hour in blocking buffer consisting of PBS, Fetal bovine serum (5 %), Bovine Serum Albumin (1 %) (Merck Millipore, A9418-10G) and Triton X-100 (0.5 %) (Merck Millipore, X100-100ML). Primary antibodies were added at dilutions listed in Table 4 in blocking buffer for 2 hours at room temperature. This was followed by 3 washes in blocking buffer and the addition of secondary antibodies ( Table 4) for 1 hour in the dark at room temperature. DAPI (ThermoFisher Scientific, D1306) was added with the secondaries at 0.1 μg/mL to visualise nuclei. Samples were washed 3 times in blocking buffer and then once with PBS. Samples were imaged using a EVOS M5000 Imaging System (ThermoFisher Scientific, AMF5000).

**Table 4.** Primary and secondary antibodies.

Target	Company, Catalog	Host	Concentration
Vimentin (Primary)	Abcam, ab45939	Rabbit	1:500
α-Smooth Muscle Actin (Primary)	Merck Millipore, A5228	Mouse	1:500
Collagen 1 (Primary)	Abcam, ab34710	Rabbit	1:500
Titin (T12) (Primary)	Fürst *et al*. (1988)	Mouse	2 μg/mL
Alexa Fluor 488 anti-Mouse IgG (Secondary)	ThermoFisher Scientific, A28175	Goat	1:500
Alexa Fluor 546 anti-Rabbit IgG	ThermoFisher Scientific, A-11035	Goat	1:500
DAPI (4′,6-Diamidino-2-Phenylindole, Dihydrochloride) (Nuclear Stain)	ThermoFisher Scientific, D1306	N/A	0.1 μg/mL

*Engineered heart tissues*

EHTs constructed with and without the addition of hiPSC-CF were removed from posts after 15 days in culture (30 days post initiation of differentiation), washed three times in PBS and fixed overnight at 4 °C in 4 % paraformaldehyde solution. A scalpel was used to longitudinally section EHTs. Sectioned pieces of EHTs were blocked overnight in blocking buffer. Primary antibodies were added in blocking buffer, at the dilutions listed in Table 4, overnight at 4°C. Samples were washed three times in blocking buffer before the addition of secondary antibodies for 4 hours in the dark at room temperature. DAPI was added with the secondaries at 0.1 μg/mL to visualise nuclei. Tissue sections were placed onto coverslips (VWR, 631-0170) and mounted onto cover slides using Hydromount Histology Mounting Media (Scientific Laboratory Supplies, NAT1324). Images were acquired using a Zeiss LSM780 Confocal laser scanning microscope equipped with a C-Apochromat 63x/1.20 W objective.

### Analysis of contractile function

EHTs constructed with and without hiPSC-CF were cultured for 15 days in EHT media (30 days after initiation of differentiation) prior to recording and analysis. 10 second videos of each EHT was recorded at 1080p/30 fps using an inverted camera with a macroscopic lens (3 EHTs with iPSC-CF, 3 EHTs without iPSC-CF). Videos were converted into tif files and analysed using the ImageJ Plugin TrackMate for displacement over time. All EHTs with contractile function were included in the analysis. Contractile function was defined as at least two macroscopically visible contractions within 15 seconds. All EHTs constructed with iPSC-CF demonstrated contractile function. Only three of the nine EHTs constructed (one of three batches, three EHTs per batch) without the addition of iPSC-CF showed contractile function. The analysis of contractile function was performed blinded to the presence or absence of hiPSC-CF. Outputs were semi-automated and included contraction duration, time to peak, relaxation time, total contraction amplitude and beat rate. For converting displacement into force, elastic bending of the pillars was assumed with a measured Young’s modulus of 3.0 MPa, yielding a conversion factor of 0.26 mN/mm.

## Results

### Generation of quiescent hiPSC-CF

**Figure 3.**
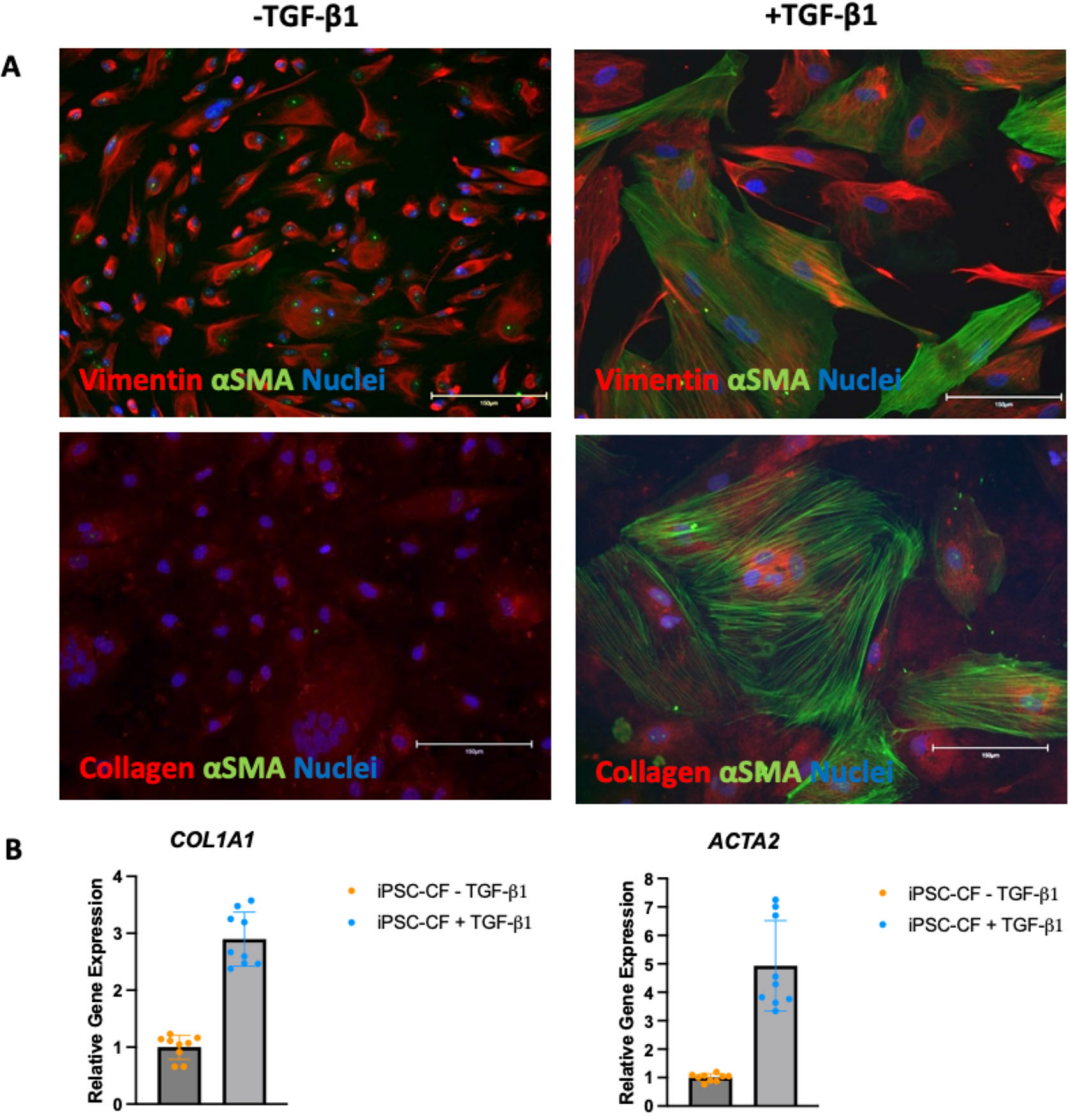
Activation state of hiPSC-CF. hiPSC-CF were cultured in the standard media (FGM3 + 10 ng/ml FGF2 + 10 μM SB 431542, ‘-TGF-β1’) and with 10 ng/mL TGF-β1 instead of the SB 431542 (‘+TGF-β1’) for 72 hours to assess activation status. A: The cells were fixed and stained for vimentin (top red), Collagen (bottom red) αSMA (green) and DAPI (blue, indicating nuclei). Scale bar = 150 microns. B: RNA was extracted, and RT-qPCR was performed on the cDNA for *ACTA2* and *COL1A1.* N = 3 (three biological samples with 3 technical replicates per sample are plotted).

Induced pluripotent stem cells were differentiated into cardiac fibroblasts using the protocol outlined in materials and methods. Following differentiation, the activation status and plasticity of the cells was assessed using immunofluorescence. The cells were cultured in the presence and absence of 10 ng/mL TGF-β1 for 72 hours. The cells were then fixed and stained for vimentin, αSMA and collagen 1 with the results shown in Figure 3A. Vimentin is a cardiac fibroblast marker irrespective of activation status whilst αSMA and collagen are predominantly expressed in activated or myofibroblasts. The hiPSC-CF cultured in the absence of TGF-β1 were positive for vimentin but negative for αSMA, indicating a quiescent cardiac fibroblast phenotype. The cells cultured without the TGF-β1 inhibitor, SB 431542, and in the presence of 10 ng/mL TGF-β1 were positive for vimentin and αSMA and showed greater expression of collagen. The gene expression of *ACTA2* (the transcript which encodes αSMA) and *COL1A1* (a transcript which encodes collagen) was assessed using RT-qPCR as markers for cardiac fibroblast activation. Expression analysis was performed on three biological samples, with three technical repeats performed for each sample. No data were excluded from the analysis. The results are shown in Figure 3B. The hiPSC-CF showed a three-fold increase in the mean expression of *COL1A1* following TGF-β1 treatment (-TGF-β1: M = 1, SD = 0.21, +TGF-β1 M = 2.9, SD = 0.47). The mean expression of *ACTA2* in the hiPSC-CF increased approximately five-fold following TGF-β1 treatment (TGF-β1: M = 1, SD = 0.13, +TGF-β1 M = 4.9, SD = 1.59).

### Macroscopic EHT structure

**Figure 4.**
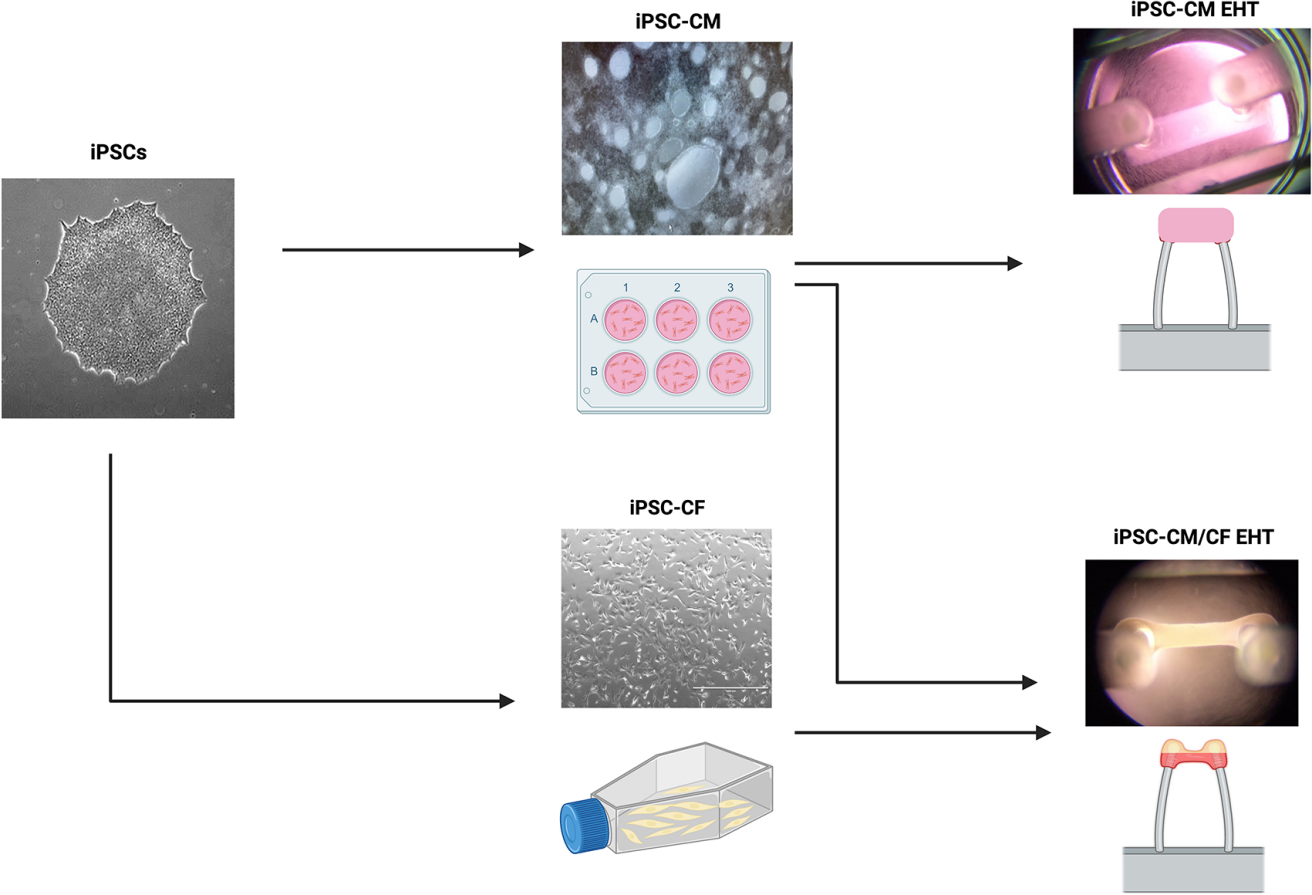
Generation of engineered heart tissues (EHTs). An outline of the workflow to generate of EHTs with and without the addition of hiPSC-CF. The EHT shown on the top right was generated without the addition of hiPSC-CF. The EHT shown on the bottom right was constructed with the addition of hiPSC-CF. Created with Biorender.com.

**Figure 5.**
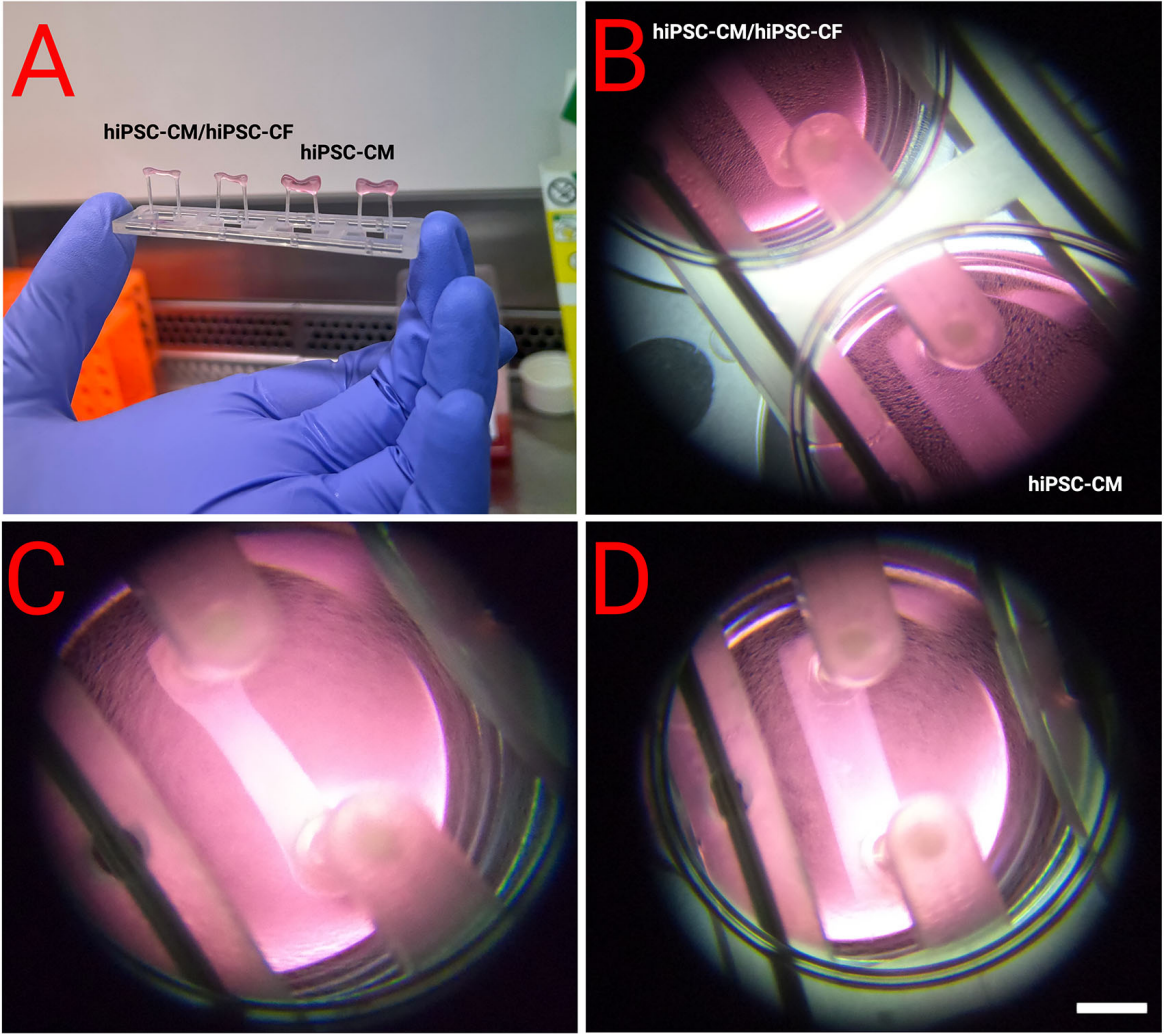
Morphology of EHTs 72 hours after construction. EHTs were constructed with and without the addition of hiPSC-CF according to the protocol outlined above. A and B show side by side comparisons. C - Close-up of an EHT with hiPSC-CF and hiPSC-CM demonstrated compaction after 72 hours, which was not observed in EHTs generated from hiPSC-CMs alone (D). Scale bar = 4 mm.

A workflow delineating the generation of EHTs from hiPSCs is displayed in Figure 4. EHTs containing hiPSC-CM and hiPSC-CF started twitching approximately 48 hours after construction, with maximal beating capacity being reached after approximately 14 days in culture. Those generated in the absence of hiPSC-CF started twitching approximately 96 hours after construction and showed diminished contractile function throughout the duration of culture. EHTs constructed with and without hiPSC-CF demonstrated marked differences in shape and structure after 72 hours in culture as shown in Figure 5. EHTs constructed with hiPSC-CF showed tightening around the silicon posts whilst hiPSC-CM-only EHTs remained more block-like throughout time in culture.

### Contractile analysis of EHTs

**Figure 6.**
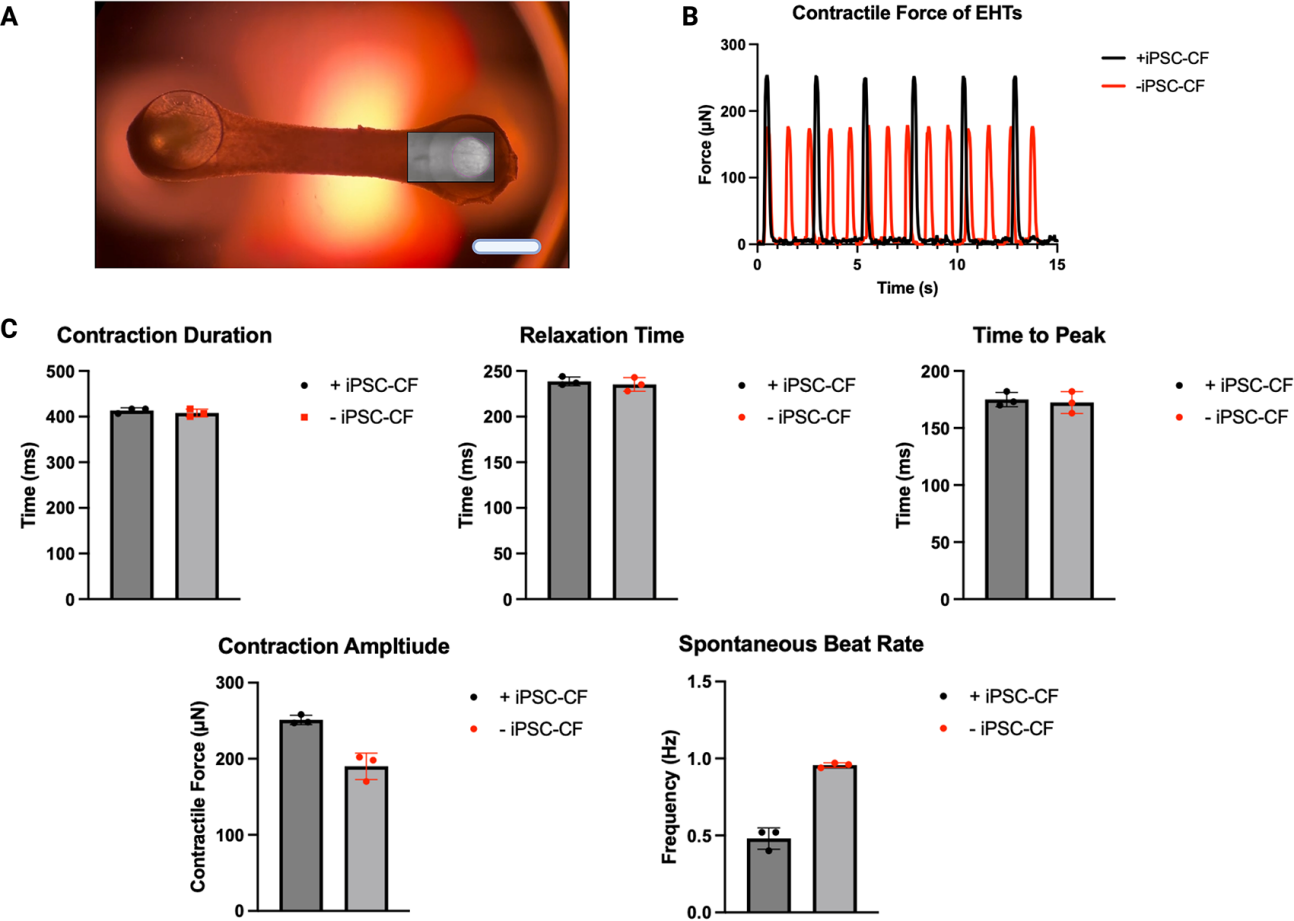
Contractile analysis of EHTs. EHTs constructed with and without the addition of hiPSC-CF were analysed to assess contractile activity. Frames of the videos of the EHTs were analysed using the ImageJ Plugin TrackMate for displacement over time, the box represents the analysis window where movement of the pillar was tracked (A). Contractile profiles were generated (a representative profile of an EHT with and without iPSC-CF is shown) (B) and used to ascertain Contractile Duration, Relaxation Time, Time to Peak, Contraction Amplitude and Spontaneous Beat Rate (C). N = 3 EHTs. Scale bar = 4 mm.

At day 30 after the initiation of differentiation (15 days in EHT form), 1080p/30 fps videos of EHTs with and without hiPSC-CF were analysed using the Open-source ImageJ plugin TrackMate ( Figure 6). All EHTs which demonstrated contractility were included in the analysis (Section 3.8). EHTs with and without hiPSC-CF demonstrated similar contraction durations and relaxation times of approximately 400 and 240 ms respectively. The mean time to peak time of the EHTs containing hiPSC-CF was similar to that of their hiPSC-CM-only counterparts at approximately 170 ms. The average contraction amplitude of the EHTs containing hiPSC-CF was approximately 30 % greater than that of the EHTs consisting of hiPSC-CM only. EHTs constructed with hiPSC-CF demonstrated a marked decrease in spontaneous beat rate.

### Composition of hiPSC-derived EHT

**Figure 7.**
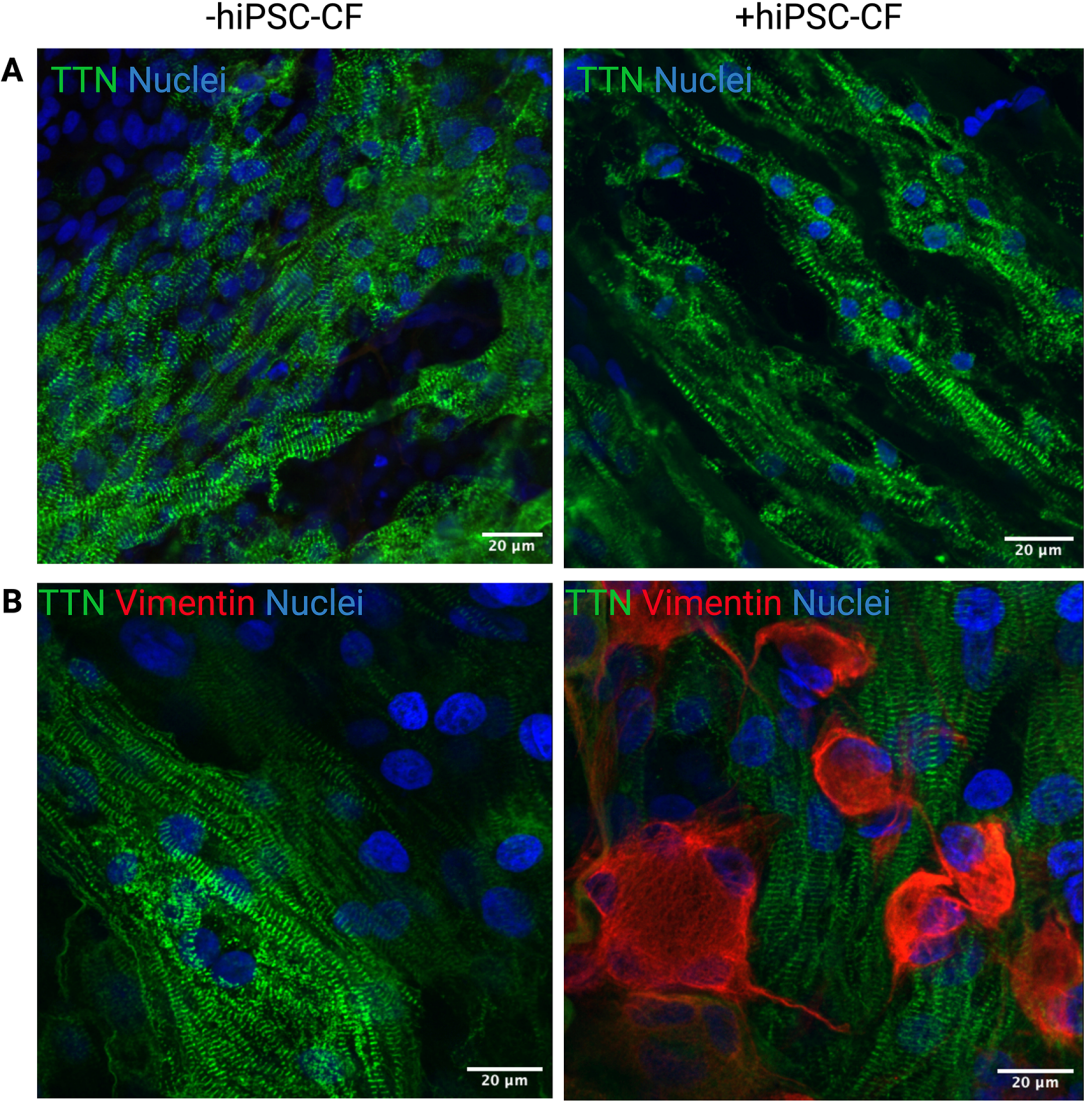
Alignment and composition of engineered heart tissues. EHTs were constructed with and without iPSC-CFs. Tissues were fixed, sectioned and stained for Titin and DAPI (A) and titin, vimentin and DAPI (B). Scale bar = 20 microns.

EHTs consisting of hiPSC-CM and hiPSC-CM/hiPSC-CF were cultured for 15 days after construction (30 days after initiation of hiPSC-CM differentiation). The tissue was fixed and sectioned using a scalpel and then stained for DAPI, titin and vimentin to assess composition and alignment ( Figure 7). Vimentin is a key marker of cardiac fibroblasts whilst titin is an integral component of the sarcomeres of cardiomyocytes. The hiPSC-CM demonstrated longitudinal alignment in EHTs constructed with and without hiPSC-CF ( Figure 7A). Cardiac fibroblasts were present in EHTs constructed with hiPSC-CF and were absent in the hiPSC-CM only tissues.

## Discussion

In this study, we described efficient and reproducible methods for the generation of hiPSC-CM and hiPSC-CF. Furthermore, we described the subsequent integration of the cells into co-culture EHTs with improved contractile function. This approach has potential to explore the complex interplay between cardiomyocyte and cardiac fibroblast signalling involved in pathophysiology. The hiPSC-CF generated were quiescent under the culture conditions outlined in the protocol, with plasticity demonstrated through the addition of the pro-fibrotic cytokine TGF-β1. EHTs constructed with the addition of hiPSC-CF exhibited anisotropic alignment, decreased beat rate, increased tissue compaction and enhanced contractile force.

The importance of non-myocyte cell types in 3D cardiac constructs is well established and has been widely discussed in previous publications ( Kofron and Mende, 2017). EHTs consisting of both hiPSC-CM and hiPSC-CF demonstrated an increase in tissue compaction following just 72 hours in culture and a decrease in beat rate after two weeks in culture. Tissue compaction plays an important role in determining contractile capability and likely occurred in the EHTs constructed with hiPSC-CF due to the regulatory function that cardiac fibroblasts have on extracellular matrix ( Rivera-Arbeláez *et al*., 2022; Thavandiran *et al*., 2013). A decrease in beat rate was observed from EHTs that were constructed with hiPSC-CF. This is consistent with previous studies that have demonstrated lower beating rate frequencies in hiPSC-CM/fibroblast co-culture models and is likely due to increased CM maturity and decreased funny current expression ( Ronaldson-Bouchard *et al*., 2018).

EHTs constructed in this study from metabolically purified populations of hiPSC-CM, without the addition of hiPSC-CF, demonstrated diminished or no contractile function. Functional and contractile EHTs consisting solely of hiPSC-CM were generated during the course of this study but with a significantly reduced rate of success. The presence of non-myocyte cell types in the tissue population has previously shown to be beneficial to the successful generation and improved contractility of 3D cardiac models ( Naito *et al*., 2006; Ravenscroft *et al*., 2016). Some studies have robustly demonstrated the successful generation of EHTs using CM populations with a purity of 92-100 % ( Mannhardt *et al*., 2016, 2020). Differences in stem cell line, hiPSC-CM differentiation, handling, and maturation may help explain contrasting success rates of hiPSC-CM only EHT construction. The further development of 3D cardiac models consisting of defined populations of cardiac myocytes and stromal cells, *e.g.*, fibroblasts, will help to reduce batch-to-batch variability and contribute to the development of more reliable *in vitro* modelling systems.

The relative ease by which hiPSCs can be genetically engineered has allowed researchers to efficiently interrogate the effects pathogenic variants have in the cardiac myocyte. The development of isogenic EHTs consisting of hiPSC-derived cardiomyocytes and cardiac fibroblasts provides a more physiological model in which mutations can be explored and presents increased opportunities for exploring variants with pathological effect in both cell types ( Zou *et al*., 2022). Furthermore, such models represent an exciting prospect for the future incorporation into pre-clinical drug screening and may eventually help relieve the current reliance on *in vivo* models.

Differences in the intrinsic electrophysiological properties of mouse cardiac myocytes have hampered efforts to model complex cardiovascular diseases. Furthering our understanding of currently enigmatic aspects of human cardiac disease, such as the dynamic interplay between cardiac fibrosis and electrical remodelling, likely requires physiological and ultimately human models, such as the EHTs applied in this study. Improvements to human *in vitro* modelling systems are required to understand the complexities of cardiac pathology and will likely aid in the reduction and/or replacement of animal models in cardiac research.

## Data Availability

Figshare: qPCR Data of iPSC-CF Activation (ACTA2, COL1A1).xlsx, https://doi.org/10.6084/m9.figshare.23639205.v1 ( Cumberland *et al*., 2023a). Figshare: Raw Images of EHTs constructed with and without iPSC-CF, https://doi.org/10.6084/m9.figshare.23978340.v1 ( Cumberland *et al*., 2023b). This project contains the following underlying data:
‐ EHT -iPSC-CM TTN.tif ‐ EHT +iPSC-CM TTN.tif ‐ EHT Vim:TTN -iPSC-CF.tif ‐ EHT Vim:TTN +iPSC-CM.tif Data are available under the terms of the Creative Commons Attribution 4.0 International license (CC-BY 4.0).
