# Supplementary material for: Generation of a human iPSC-derived cardiomyocyte/fibroblast engineered heart tissue model
Source: F1000Res. 2024 Feb 12;12:1224. Originally published 2023 Sep 27. [Version 2] doi: 10.12688/f1000research.139482.2 (PMC10828555; doi:10.12688/f1000research.139482.2)
Supplement: Peer review history

# Reviewer report: Reviewer response for version 2

**DOI:** 10.5256/f1000research.162401.r246071

**Published:** 2024-02-14

**Authors:** Emma Louise Robinson

**Affiliations:** 1 University of Colorado Denver, Denver, Colorado, USA

Thank you for addressing my prior comments. 

 Congratulations!

Is the rationale for developing the new method (or application) clearly explained?

Yes

Is the description of the method technically sound?

Yes

Are the conclusions about the method and its performance adequately supported by the findings presented in the article?

Partly

If any results are presented, are all the source data underlying the results available to ensure full reproducibility?

Yes

Are sufficient details provided to allow replication of the method development and its use by others?

Yes

Reviewer Expertise:

heart failure, epigenetics, signaling

I confirm that I have read this submission and believe that I have an appropriate level of expertise to confirm that it is of an acceptable scientific standard.

# Reviewer report: Reviewer response for version 1

**DOI:** 10.5256/f1000research.152758.r221607

**Published:** 2024-01-30

**Authors:** Mike Dodd

**Affiliations:** 1 Centre for Health and Life Sciences, Coventry University (Ringgold ID: 2706), Coventry, England, UK

Cumberland and colleagues have produced a really good protocol on the generation of more mature EHT using a combination of iPSC- cardiomyocytes and fibroblasts. It flows really well and I find it easy to follow, supported by good figures and details of reagents.

 One of the areas where iPSC-CM models still need to improve is in the metabolic maturity to better replicate the adult heart. With the co-culturing of cardiac fibroblast with cardiomyocytes in this EHT system it would be useful to know whether there is an improvement in metabolic markers. Whilst something like Clarke electrodes for measuring metabolic rate would be excellent, I would like to see some qPCR data on metabolic genes. Some of the key genes in the transition between fetal (immature) and adult cardiac models would be increase GLUT4, PPARA and fatty acid oxidation gene expression and possibly decrease in pyruvate kinase 2 and hexokinase 1. I would be interested to see the differences in these profiles between the two EHT models, to see if the CF are increasing metabolic maturity. Otherwise this is an excellent piece of work.

Is the rationale for developing the new method (or application) clearly explained?

Yes

Is the description of the method technically sound?

Yes

Are the conclusions about the method and its performance adequately supported by the findings presented in the article?

Yes

If any results are presented, are all the source data underlying the results available to ensure full reproducibility?

Yes

Are sufficient details provided to allow replication of the method development and its use by others?

Yes

Reviewer Expertise:

cardiac metabolism, epigenetics, diabetes and hypoxia.

I confirm that I have read this submission and believe that I have an appropriate level of expertise to confirm that it is of an acceptable scientific standard.

# Reviewer report: Reviewer response for version 1

**DOI:** 10.5256/f1000research.152758.r221605

**Published:** 2023-11-22

**Authors:** Emma Louise Robinson

**Affiliations:** 1 University of Colorado Denver, Denver, Colorado, USA

Congratulations to the authors on a thorough and well-described methods paper on how to generate an engineered heart tissue comprised of iPSC-derived CMs and CFs. The text and figures are clear and the article flows very well.

 One suggestion is to highlight, in a line or two, the novelty of this EHT system over other similar EHT methods published.

 Another is to comment on the omission of demonstrating responsiveness of the iPSC-CMs to pathological stimuli, such as isoproterenol or endothelin-1 increasing ANF and BNP production, for example. If this EHT system is to be used to model pathological remodeling and heart failure, this would have been nice. I am unsure how much time and effort it would take to add this data or whether the authors have some relevant data already along these lines. There is a nice demonstration of the activation of iPSC-CFs to TGF-Beta.

Is the rationale for developing the new method (or application) clearly explained?

Yes

Is the description of the method technically sound?

Yes

Are the conclusions about the method and its performance adequately supported by the findings presented in the article?

Partly

If any results are presented, are all the source data underlying the results available to ensure full reproducibility?

Yes

Are sufficient details provided to allow replication of the method development and its use by others?

Yes

Reviewer Expertise:

heart failure, epigenetics, signaling

I confirm that I have read this submission and believe that I have an appropriate level of expertise to confirm that it is of an acceptable scientific standard, however I have significant reservations, as outlined above.

# Reviewer report: Reviewer response for version 1

**DOI:** 10.5256/f1000research.152758.r210539

**Published:** 2023-10-13

**Authors:** Claudia Crocini

**Affiliations:** 1 Charite' Berlin, Berlin, Germany

The work by Cumberland *et al.*, describes a protocol to generate the EHT using cardiomyocytes and cardiac fibroblasts derived from the same hiPSCs.

 The work is not particularly new but is well-described.

 My comments:

1. I would like to see the force expressed as mN/mm2 measuring the diameter of EHTs. I know that the EHT community doesn't do it but the physiology community does not agree. The raw values could be showed in a table or in the supplement.
2. Figure 7: It would be better to see the EHTs with the vimentin and TTN signals splitted (instead of the supplement). Also a maximum intensity projection of a z-stack could help to avoid seeing blue nuclei with very faint green/red signal.

Is the rationale for developing the new method (or application) clearly explained?

Yes

Is the description of the method technically sound?

Yes

Are the conclusions about the method and its performance adequately supported by the findings presented in the article?

Yes

If any results are presented, are all the source data underlying the results available to ensure full reproducibility?

Yes

Are sufficient details provided to allow replication of the method development and its use by others?

Yes

Reviewer Expertise:

cardiac biology, imaging, optogenetics

I confirm that I have read this submission and believe that I have an appropriate level of expertise to confirm that it is of an acceptable scientific standard, however I have significant reservations, as outlined above.
